# Percutaneous Intervention in External Outflow Graft Obstruction of Magnetically Levitated Left Ventricular Assist Device: Long‐Term Follow‐Up and Quality of Life

**DOI:** 10.1111/aor.70144

**Published:** 2026-04-26

**Authors:** Anna Huang, Julia Stein, Vanessa I. T. Zwaans, Carla L. Schuering, Gaik Nersesian, Christoph Hoermandinger, Markus Müller, Johanna Mulzer, Stephan Dreysse, Philipp Stawowy, Christoph T. Starck, Joerg Kempfert, Evgenij V. Potapov, Volkmar Falk, Leonhard Wert

**Affiliations:** ^1^ Department of Cardiothoracic and Vascular Surgery Deutsches Herzzentrum der Charité Berlin Germany; ^2^ Charité – Universitätsmedizin Berlin, Corporate Member of Freie Universität Berlin and Humboldt‐Universität zu Berlin Berlin Germany; ^3^ Department of Cardiology, Angiology and Intensive Care Medicine Deutsches Herzzentrum der Charité Berlin Germany; ^4^ DZHK (German Center for Cardiovascular Research), Partner Site Berlin Berlin Germany; ^5^ Department of Health Sciences and Technology ETH Zürich Zurich Switzerland

**Keywords:** biodebris, external outflow graft obstruction, HeartMate 3, left ventricular assist device, outflow graft, outflow graft compression, outflow graft tamponade

## Abstract

**Objectives:**

External compression of the outflow graft causing obstruction (eOGO) is a potentially lethal complication in patients on long‐term mechanical circulatory support with the HeartMate 3 (HM3, Abbott) left ventricular assist device (LVAD). This complication results from the build‐up of gelatinous substance between the bend relief and outflow graft and can be resolved by percutaneous intervention, surgery, or transplantation. This single‐centre follow‐up study evaluated the suitability of percutaneous intervention as a treatment strategy and long‐term outcomes of eOGO patients in terms of laboratory, LVAD, and quality‐of‐life parameters.

**Methods:**

On October 31, 2024, a search of the implantation centre's electronic database identified HM3 patients diagnosed with eOGO. Individual patient data concerning 31 cases was analyzed. A quality‐of‐life survey was conducted using the short version of the Kansas City Cardiomyopathy Questionnaire (KCCQ‐12).

**Results:**

The patient cohort had a median support time to eOGO diagnosis of 1219 days [976, 1917] and a post‐treatment follow‐up of 686 days [447, 1003]. 64.5% of patients (*n* = 20) underwent percutaneous intervention showing immediate LVAD flow improvement of 0.5 L/min post‐intervention (*p* = 0.04). Of eight post‐interventional survey respondents, 62.5% (*n* = 5) were assigned a fair‐excellent health status according to the KCCQ‐12.

**Conclusions:**

Percutaneous intervention is a suitable treatment strategy for eOGO, resolving low flow and providing satisfactory long‐term quality of life outcomes. Given the increasing eOGO incidence after 1 year of support and overall mortality of 29.0%, clinicians should remain on high alert for this complication. We suggest computed tomography (CT) imaging be considered early on when eOGO is suspected.

## Graphical abstract

eOGO is an uncommon adverse event in patients on long‐term LVAD support. Thirty‐one eOGO patients were identified in a single‐centre HM3 implantation program. Twenty patients underwent percutaneous stenting. This treatment resulted in immediate LVAD flow improvement and yielded satisfactory quality‐of‐life results, with 62.5% patients assigned a fair‐excellent health status per the KCCQ‐12.

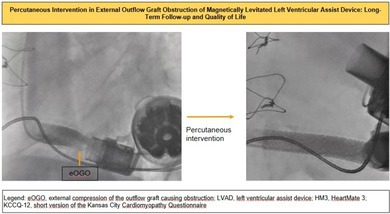

**Abbreviations**
AP: anteroposterior
aPTT: activated partial thromboplastin clotting time
CMP: cardiomyopathy
CT: computed tomography
DCMP: dilated cardiomyopathy
eOGO: external compression of the outflow graft causing obstruction
HM3: HeartMate 3
ICMP: ischaemic cardiomyopathy
INR: international normalized ratio
KCCQ‐12: short version of the Kansas City Cardiomyopathy Questionnaire
LDH: lactate dehydrogenase
LVAD: left ventricular assist device
LVEF: left ventricular ejection fraction
NICMP: non‐ischaemic cardiomyopathy
PA: percutaneous angiography
PET: polyethylene terephthalate

## 1 Introduction

The HeartMate 3 (HM3; Abbott) is a fully magnetically levitated centrifugal‐flow left ventricular assist device (LVAD) which has improved the outcome of patients with advanced heart failure [1]. Compared to its predecessors, the axial‐flow HeartMate 2 (HM2; Abbott) and the HVAD (Medtronic) LVAD system, the design of the HM3 system improves haemocompatibility, reducing the risk of thromboembolic events [2, 3].

**FIGURE 1:**
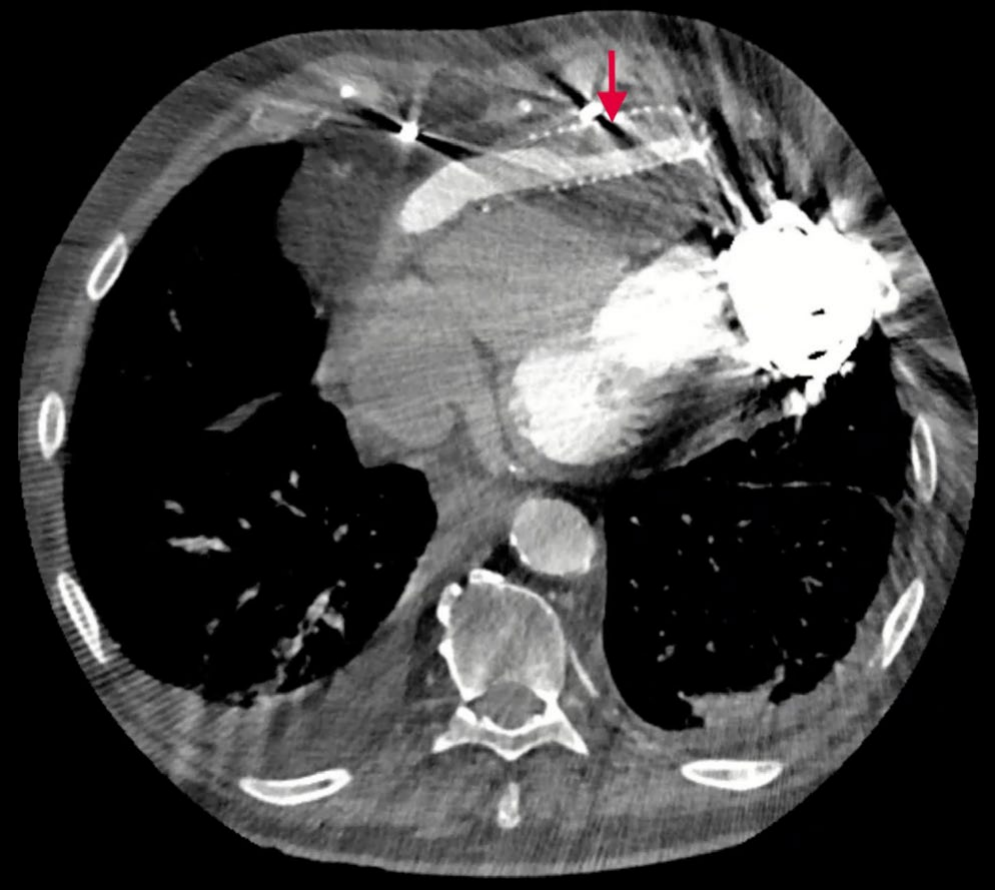
CT‐angiography image of a patient with eOGO (red arrow). *CT*, computed tomography; *eOGO*, external compression of the outflow graft causing obstruction. [Color figure can be viewed at wileyonlinelibrary.com]

Despite these advancements, external compression of the outflow graft causing obstruction (eOGO) has emerged as a serious complication in long‐term HM3 support patients, with prevalences of up to 33% and incidences of up to 9.1% at 5 years of HM3 support being reported [4, 5, 6]. Although uncommon, eOGO is a potentially fatal complication that is characterized by extrinsic outflow graft compression due to an accumulation of gelatinous substance between the outflow graft and bend relief. Proteinaceous blood components permeate the porous polyethylene terephthalate (PET) outflow graft, but then become trapped by the impermeable polytetrafluoroethylene (Gore‐Tex) bend relief. Gradually, the proteinaceous components accumulate and form a gelatinous substance in the space between the bend relief and outflow graft that compresses the outflow graft causing luminal narrowing and reduced flow rate (Figure 1) [6, 7, 8, 9]. In response to this, Abbott issued an Urgent Field Safety Notice in February 2024, announcing upcoming design modifications for the HM3 intended to reduce the gelatinous build‐up between the outflow graft and the bend relief, potentially preventing future cases of eOGO [10].

Currently, the lack of standardized screening guidelines complicates efforts to accurately assess the impact of eOGO on long‐term HM3 support patients [11]. Cases are often diagnosed by coincidence or are not detected [6, 7, 12]. Therefore, current estimates are likely being underestimated owing to the large number of undiagnosed cases. Due to many studies performing eOGO diagnosis by computed tomography (CT) assessment, we suggest the use of the three‐tier grading system as outlined in the 2024 review by Huang et al. [11].

Treatment strategies for eOGO include surgery, heart transplantation, or percutaneous stenting.

**FIGURE 2:**
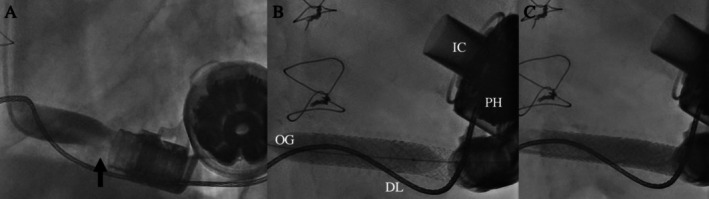
Percutaneous angiography of a patient with eOGO. (A) Preinterventional angiography of the outflow graft with impaired blood flow due to eOGO (black arrow). (B) Stent insertion and balloon dilatation. (C) Postinterventional angiography of the outflow graft with improved flow. *DL*, driveline; *eOGO*, external compression of the outflow graft causing obstruction; *IC*, inflow cannula; *OG*, outflow graft; *PH*, pump housing.

Percutaneous stenting (Figure 2) is frequently the treatment of choice for eOGO and favorable in terms of its lower risk profile when compared to surgical methods [4, 5, 6, 7, 8, 13, 14, 15, 16, 17, 18, 19, 20, 21, 22, 23].

At our implantation centre, percutaneous stenting is also the preferred treatment strategy, with the following approach to periprocedural management: Stent sizing is based on CT images and the experience of the intervening cardiologist. LVAD patients at our clinic are managed with an antiplatelet drug, either acetylsalicylic acid or clopidogrel, and additionally phenprocoumon. Periprocedural bridging is done with heparin with no additional anticoagulation regimen.

Existing literature evaluates the efficacy and short‐term outcomes of endovascular treatment of eOGO, with reported post‐interventional mortality of 9.5% 12% and follow‐up time of 90 days to 3.9 months. However, long‐term outcomes remain unreported [23, 24, 25].

To evaluate the long‐term outcomes of stented eOGO patients, we conducted a long‐term follow‐up study including a quality‐of‐life (QoL) survey of eOGO patients at our implantation centre who underwent percutaneous stenting.

## 2 Patients and Methods

### 2.1 Study Design

This is a single‐centre, retrospective study for which data was collected from patients supported with an HM3 LVAD. The institutional review board or equivalent ethics committee of the authors' institutions approved the study protocol and publication of data. The study was approved by the institutional review board at the Charité—Universitätsmedizin Berlin (approval number: EA4/150/25) on July 7, 2025. The patients provided informed written consent for the publication of the study data.

### 2.2 Patient Inclusion Criteria

An electronic search of the implantation centre's patient database was performed on October 31, 2024, with following search words: “OGO”, “eOGO”, “outflow graft” (in German), “outflow graft stenosis” (in German) and “external outflow graft stenosis” (in German). The resulting patient list was then screened for inclusion criteria.

Patient inclusion criteria were as follows:

1. Implantation of the HM3 LVAD.
2. Confirmation of eOGO diagnosis intraoperatively, by CT imaging or by percutaneous angiography (PA).

Patients were excluded if there was no definitive eOGO diagnosis, as were those with an intraluminal thrombus, a twisted or kinked outflow graft without additional eOGO.

### 2.3 Data Extraction

Individual patient data was extracted from the electronic patient database by the first author. The extracted data included: patient birthdate and sex, cardiac pathology, therapy target, date of LVAD implantation, surgical access for LVAD implantation, date of eOGO diagnosis, device flow rate at admission and discharge, motor power at admission and discharge, serum lactate dehydrogenase at admission and discharge, INR at admission and discharge, CT results, echocardiography parameters, percutaneous angiography results, surgical methods to treat eOGO, type of stent used in percutaneous intervention, date of last contact or date of death, date of heart transplantation.

### 2.4 Quality‐of‐Life Assessment

Quality of life was assessed using the short version of the Kansas City Cardiomyopathy Questionnaire (KCCQ‐12). We obtained permission for usage and reproduction from the copyright holder. The KCCQ‐12 measures physical limitations, symptom burden and frequency, quality of life and social limitations [26] (Figure S1). Each subdomain is scored from 0 to 100, with the mean of these yielding the summary score that categorizes patients to a corresponding health status: 0–24 (very poor to poor), 25–49 (poor to fair), 50–74 (fair to good), and 75–100 (good to excellent) [27, 28, 29].

### 2.5 Statistical Analysis

Available data was extracted from eligible patients' history and included in the statistical analysis.

Categorical variables are presented as N (%) and continuous variables are summarized as mean ± SD or median with interquartile range [IQR]. Continuous variables (LVAD flow rate and motor power) were compared using Student's *t*‐test, with a two‐sided *p* < 0.05 being considered statistically significant. Descriptive statistical analysis was performed using the data analysis tool R, Version 4.02.

A cumulative incidence curve was plotted for all eOGO patients and a Kaplan–Meier analysis was performed for stented patients.

## 3 Results

### 3.1 Demographics and Baseline Characteristics

Thirty‐one HM3 patients diagnosed with eOGO were identified in the database of the implantation centre.

Individual patient data with baseline characteristics as well as surgical access for LVAD implantation is fully described in Tables S1 and S2.

Twenty‐four patients (77.4%) were male. The mean age was 57 ± 12 years, and the median LVAD support duration before eOGO diagnosis was 1219 days [976, 1917].

Eighteen patients (58.1%) suffered from ischaemic and twelve patients (38.7%) from dilated cardiomyopathy. One patient (3.2%) had non‐ischaemic cardiomyopathy without further subtype classification.

The common therapy targets for HM3 implantation were destination therapy (*n* = 23, 74.2%) and bridge to transplant (*n* = 8, 25.8%).

Full sternotomy was performed in 26 patients (83.9%), compared to lateral thoracotomy in 5 patients (16.1%).

### 3.2 Incidence and Clinical Presentation of eOGO

**FIGURE 3:**
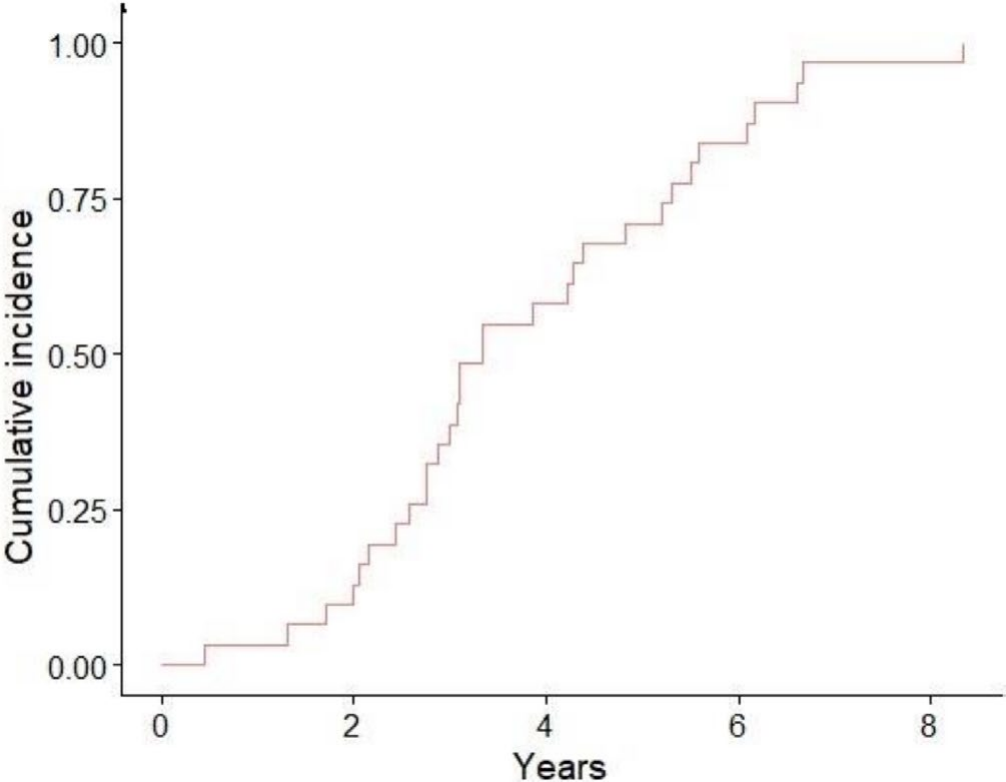
Cumulative incidence graph of eOGO in HM3 patients over 8.3 years. *eOGO*, external compression of the outflow graft causing obstruction; *HM3*, HeartMate 3. [Color figure can be viewed at wileyonlinelibrary.com]

Within the eOGO patient cohort, 3.2% patients developed eOGO within 1 year of HM3 support, 9.7% within 2 years, 35.5% within 3 years, 58.1% within 4 years, 71.0% within 5 years, 83.9% within 6 years, 96.8% within 7 years, with the last reported case at 8.3 years (Figure 3, Table S3).

In terms of clinical symptoms, ten patients (32.3%) presented with dyspnoea, six patients (19.4%) with low‐flow alerts, five patients (16.1%) with hydropic decompensation, three patients (9.7%) with angina pectoris and one patient (3.2%) each with anemia, syncope or fatigue. Seven patients (22.6%) were asymptomatic with eOGO being diagnosed incidentally during routine diagnostic assessments (for heart transplantation evaluation) or during diagnostic tests carried out for an unrelated health complaint (Tables S4 and S5).

### 3.3 Diagnosis and Treatment

CT findings (Video S1) were available for all 31 patients according to which eOGO severity was classified following the guidelines outlined by Huang et al. [11]. Nine eOGO cases (29.0%) were classified as mild, another twelve (38.7%) as moderate, and ten (32.3%) as severe (Table S5).

Subsequently, the patients were assigned to an eOGO treatment group based on the treatment option they received: watchful waiting, stenting, surgery, or heart transplantation. If patients received multiple treatments, they were assigned according to the last treatment option they received (such as heart transplantation after prior percutaneous intervention).

**FIGURE 4:**
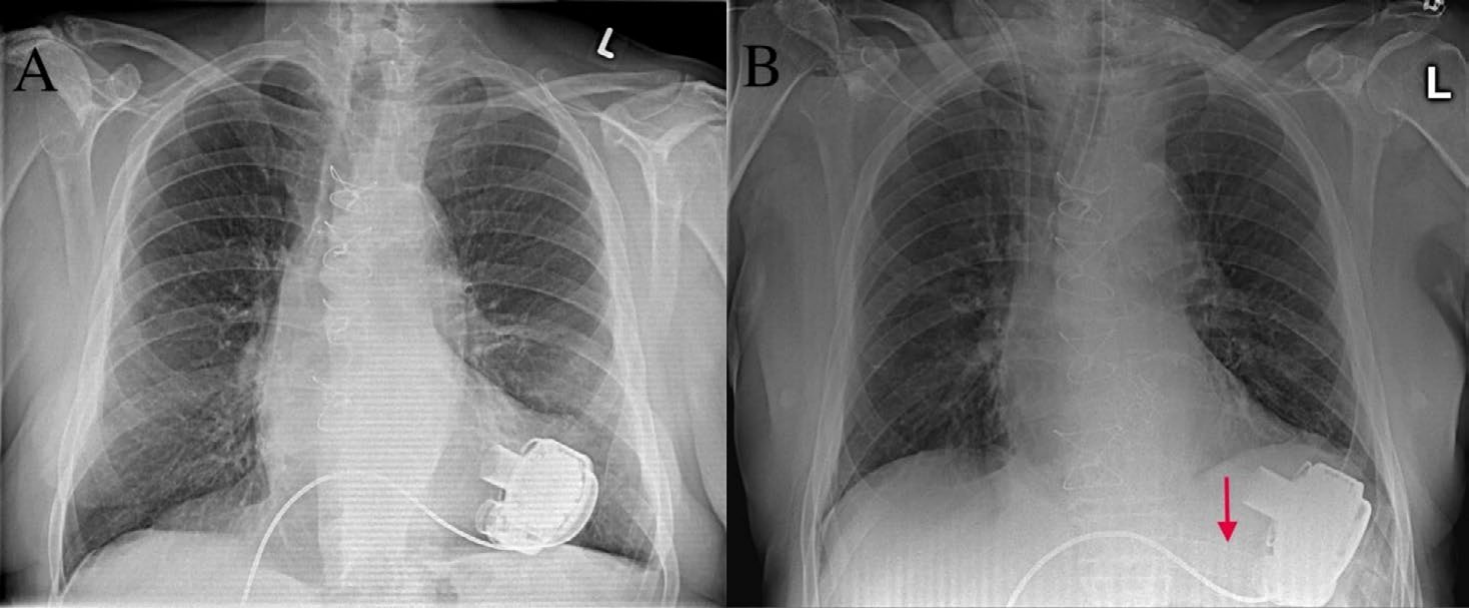
X‐ray images in AP view of a patient with eOGO. (A) Preinterventional X‐ray image showing placement of HM3 device. (B) Postinterventional X‐ray image showing stented outflow graft (red arrow). *AP*, anteroposterior; *eOGO*, external compression of the outflow graft causing obstruction; *HM3*, HeartMate 3. [Color figure can be viewed at wileyonlinelibrary.com]

Nine patients (29.0%) were assigned to the watchful waiting group; these were all classified as either mild or moderate eOGO cases. The majority, 20 patients (64.5%), received a percutaneous intervention (Figure 4), with one patient (3.2%) each being surgically treated and receiving a heart transplantation (Table S5).

Overall, we report a median follow‐up time after treatment of 686 days [447, 1003] and a mortality of 29.0% (nine patients).

### 3.4 Percutaneous Intervention and Outcome

**FIGURE 5:**
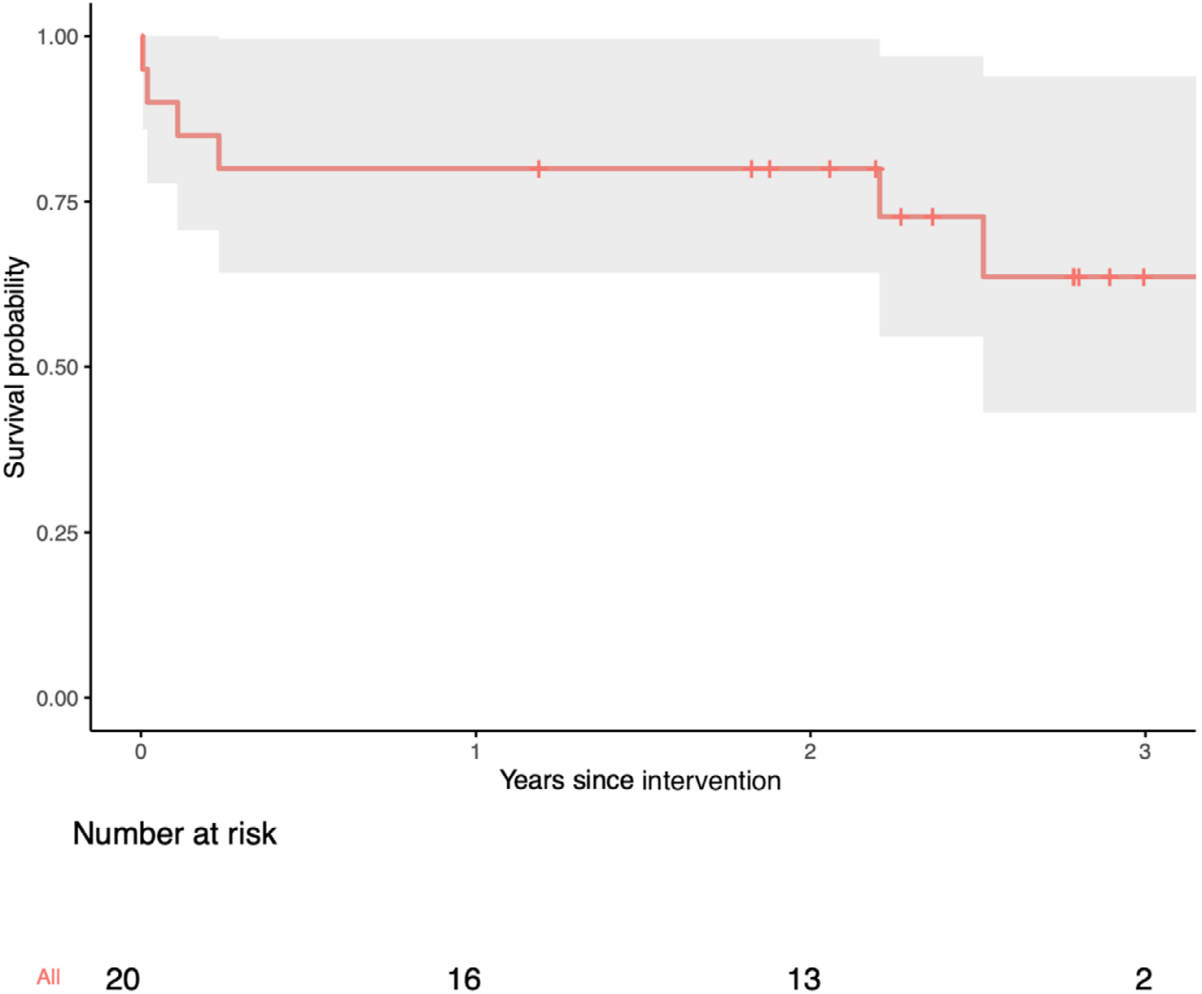
Survival probability of eOGO patients treated with percutaneous stenting with 95% CI indicated by shading. Follow‐up time in years. eOGO, external compression of the outflow graft causing obstruction. [Color figure can be viewed at wileyonlinelibrary.com]

In the patient cohort which received percutaneous stenting as eOGO treatment strategy (Video S2, Table S6), mean LVAD flow improved from 4.4 L/min to 4.9 L/min (confidence interval: −0.88–0.03; *p* = 0.04) with detailed laboratory and HM3 device parameters described in Tables S7 and S8. The median follow‐up time was 1474 days [1103, 1952] with an overall mortality of 30.0% (six patients) and 30‐day mortality of 10.0% (two patients) (Figure 5, Table S9).

### 3.5 Quality‐Of‐Life Assessment

Surviving patients were interviewed by telephone using the KCCQ‐12 for a quality‐of‐life assessment following eOGO and treatment thereof. In total, 15 patients were available for an interview; of these, eight patients were stented (Table S10). Of eight postinterventional patients, one patient scored less than 24 points, corresponding to a very poor to poor health status. Another two patients scored between 25 and 49 points, which corresponds to a poor to fair health status. Three patients received a score between 50 and 74 points and were assigned a fair to good health status. Lastly, two patients scored 75 or more points, corresponding to a good to excellent health status [27].

## 4 Discussion

This study reports on 31 eOGO cases, the largest single‐centre cohort of HM3 patients diagnosed with eOGO to date.

Findings concerning the marked rise in eOGO incidence in HM3 patients after 1 year on LVAD support are consistent with results from previous studies: Wert et al. reported an overall eOGO prevalence of 3.0% with a rising incidence after 1 year on HM3 support in a multicentre study [6]. Our results show an increase in cumulative incidence from 3.2% to 9.7% between 1 and 2 years on HM3 support.

Symptoms at presentation were heterogeneous, with dyspnoea and low‐flow alarms being the most common, aligning with prior reports [6, 11]. Over 20% of patients were asymptomatic, with incidental eOGO diagnosis during unrelated diagnostic assessments. These findings emphasize the need for clinical vigilance for this complication, even if patients present atypically or asymptomatically.

CT imaging was used in all cases, highlighting its important role in the differential diagnosis process. Our results show that the watchful waiting treatment group (*n* = 9) only consisted of patients with mild to moderate eOGO. Two patients within this treatment group expired (22.2%) suggesting watchful waiting may be a suitable approach for selected patients.

Percutaneous stenting was the most common treatment and showed favorable short‐ and long‐term results, including statistically significant LVAD flow improvement, 30.0% overall mortality and 10.0% 30‐day mortality. These outcomes support previous reports describing endovascular stenting as an effective and relatively low‐risk treatment for eOGO [18, 24, 25].

Only one patient underwent surgical revision of the outflow graft, highlighting both the technical complexity and high risk profile associated with open surgery in HM3 patients. Such strategies should be reserved for patients if percutaneous stenting fails.

The quality‐of‐life survey showed variable outcomes (Table S10), with 62.5% of stented patients receiving a ‘fair‐good’ or ‘good‐excellent’ health status. Notably, over half of the interviewed patients (*n* = 8) reported feeling limitations regarding showering/bathing; this is because showering with changing of the wound dressing takes place once weekly and bathing is not feasible. Encouragingly, 75.0% of stented patients did not report signs of oedema. However, scoring of fatigue and dyspnoea was more heterogeneous, which shows that despite restored device function, patients still experience a high symptom burden.

Abbott recently introduced modified bend relief to prevent gelatinous build‐up, now in use in the United States and soon available in Europe [10]. Some implantation centres perforate the bend relief of the HM3 device prior to implantation to mitigate eOGO risk [30]. Additionally, other manufacturers, such as Corvion, are incorporating a fenestrated bend relief into next‐generation LVADs [31]. However, we anticipate that these measures may only delay the onset of eOGO rather than provide a definitive solution to the underlying issue.

## 5 Conclusions

eOGO is a potentially life‐threatening complication in HM3 LVAD patients on prolonged support occurring due to build‐up of gelatinous substance between the bend relief and outflow graft. This follow‐up study assessed the outcome of 31 eOGO patients. eOGO diagnosis was confirmed by CT imaging, and patients mostly underwent percutaneous intervention. Percutaneous stenting is a suitable eOGO treatment strategy, resulting in immediate improvement of LVAD parameters and yielding favorable quality‐of‐life outcomes for most patients. Due to the lower risk profile in comparison to surgery, we believe that percutaneous intervention will be established as the preferred treatment method for eOGO in the future.

Routine long‐term imaging surveillance may play a critical role in detecting eOGO early on, particularly as the cohort of long‐term HM3 patients continues to grow. CT imaging should be considered early in the diagnostic workup when eOGO is suspected. Future prospective studies are needed to better define pathophysiology and optimal screening strategies.

## Author Contributions

Statistical analysis: Anna Huang, Julia Stein. Concept/design, and drafting of the article: Anna Huang, Leonhard Wert. Critical revision of the article: Julia Stein, Vanessa I.T. Zwaans, Carla L. Schuering, Gaik Nersesian, Christoph Hoermandinger, Markus Müller, Johanna Mulzer, Stephan Dreysse, Philipp Stawowy, Christoph T. Starck, Joerg Kempfert, Volkmar Falk, and Evgenij V. Potapov.

## Disclosure

Institutional Review Board Approval: EA4/150/25 (7th of July 2025).

## Conflicts of Interest

Christoph T. Starck: Payment to his institution related to his activity as speaker fees, honoraria, consultancy, advisory board fees, investigator, committee member of AngioDynamics, Abiomed, Medtronic, Spectranetics, Biotronik, LivaNova (Sorin) and Cook Medical and departmental or institutional research funding from Cook Medical. Joerg Kempfert: Grants or contracts from any entity: Edwards, LivaNova. Payment or honoraria for lectures, presentations, speakers bureaus, manuscript writing or educational events: Edwards, Medtronic, Abbott, LivaNova, CryoLife. Leadership or fiduciary role in other board, society, committee or advocacy group, paid or unpaid: TC EACTS, ECSC Board, ISMICS Board. Volkmar Falk: Grants or contracts from any entity: Medtronic GmbH, Biotronik SE & Co., Abbott GmbH & Co. KG, Boston Scientific, Edwards Lifesciences, Berlin Heart, Novartis Pharma GmbH, JOTEC/Cryolife GmbH, LivaNova, Zurich Heart. I hereby declare that I have relevant (institutional) financial activities outside the submitted work with the mentioned commercial entities in relation to educational grants (including travel support), fees for lectures and speeches, fees for professional consultation, research and study funds. Evgenij V. Potapov: Consulting fees: Abbott (institutional grants), Medtronic (institutional grants), Abiomed (institutional grants). Payment or honoraria for lectures, presentations, speakers bureaus, manuscript writing or educational events: Abbott (institutional grants), Medtronic (institutional grants), Abiomed (institutional grants). Support for attending meetings and/or travel: Abbott (institutional grants), Medtronic (institutional grants), Abiomed (institutional grants). Participation on a Data Safety Monitoring Board or Advisory Board: Abbott, Medtronic.

The other authors declare no conflicts of interest.

## Supporting information


**Figure S1:** KCCQ‐12 questionnaire used for quality‐of‐life assessment. *KCCQ‐12*, short version of the Kansas City Cardiomyopathy Questionnaire.
**Table S1:** Demographic and baseline characteristics of HM3 patients with eOGO at the implantation centre. Data on surgical access for implantation was available for nine patients and was taken into account for the percentage calculations. HM3, HeartMate 3; eOGO, external compression of the outflow graft causing obstruction.
**Table S2:** Baseline characteristics of individual patients. HM3, HeartMate 3; CMP, cardiomyopathy; ICMP, ischaemic cardiomyopathy; DCMP, dilated cardiomyopathy; NICMP, non‐ischaemic cardiomyopathy; eOGO, external compression of the outflow graft causing obstruction.
**Table S3:** Incidence table of patients on HM3 support who developed eOGO. HM3, HeartMate 3; eOGO, external compression of the outflow graft causing obstruction.
**Table S4:** Symptoms displayed by eOGO patients. eOGO, external compression of the outflow graft causing obstruction.
**Table S5:** Clinical symptoms, diagnostic assessment and treatment of individual patients. CT, computed tomography; eOGO, external compression of the outflow graft causing obstruction.
**Table S6:** Stent types used for eOGO patients during percutaneous intervention. Unavailable information is marked as such. eOGO, external compression of the outflow graft causing obstruction.
**Table S7:** Laboratory parameters of eOGO patients who received percutaneous intervention. INR, International Normalized Ratio; LVAD, left ventricular assist device; eOGO, external compression of the outflow graft causing obstruction; LDH, lactate dehydrogenase; Hb, hemoglobin; aPTT, activated partial thromboplastin clotting time.
**Table S8:** LVAD parameters of eOGO patients who received percutaneous intervention. LVAD, left ventricular assist device; eOGO, external compression of the outflow graft causing obstruction.
**Table S9:** Follow‐up times and outcomes of eOGO patients with different treatments strategies. eOGO, external compression of the outflow graft causing obstruction.
**Table S10:** Results from quality‐of‐life assessment of available eOGO patients post‐treatment. The KCCQ‐12 was used to interview patients with the question number and corresponding score outlined as well as the resulting health status. eOGO, external compression of the outflow graft causing obstruction, KCCQ‐12, short version of the Kansas City Cardiomyopathy Questionnaire; Q, question.

**Video S1:** Computed tomography angiography of external outflow graft obstruction.

**Video S2:** Patient with external outflow graft obstruction undergoing percutaneous angiography with stenting. (A) Preinterventional angiography of the compressed outflow graft. (B) Stent placement. (C) Balloon dilatation. (D) Stented outflow graft showing improved flow.

## Data Availability

The data underlying this article are available in the article and in its online Supporting Information.
