# Supplementary material for: Percutaneous Intervention in External Outflow Graft Obstruction of Magnetically Levitated Left Ventricular Assist Device: Long‐Term Follow‐Up and Quality of Life
Source: Artif Organs. 2026 Apr 26;50(7):1060–6. doi: 10.1111/aor.70144 (PMC13397190; doi:10.1111/aor.70144)
Supplement: Supplementary file 1 — Figure S1: KCCQ‐12 questionnaire used for quality‐of‐life assessment. KCCQ‐12, short version of the Kansas City Cardiomyopathy Questionnaire. Table S1: Demographic and baseline characteristics of HM3 patients with eOGO at the implantation centre. Data on surgical access for implantation was available for nine patients and was taken into account for the percentage calculations. HM3, HeartMate 3; eOGO, external compression of the outflow graft causing obstruction. Table S2: Baseline characteristics of individual patients. HM3, HeartMate 3; CMP, cardiomyopathy; ICMP, ischaemic cardiomyopathy; DCMP, dilated cardiomyopathy; NICMP, non‐ischaemic cardiomyopathy; eOGO, external compression of the outflow graft causing obstruction. Table S3: Incidence table of patients on HM3 support who developed eOGO. HM3, HeartMate 3; eOGO, external compression of the outflow graft causing obstruction. Table S4: Symptoms displayed by eOGO patients. eOGO, external compression of the outflow graft causing obstruction. Table S5: Clinical symptoms, diagnostic assessment and treatment of individual patients. CT, computed tomography; eOGO, external compression of the outflow graft causing obstruction. Table S6: Stent types used for eOGO patients during percutaneous intervention. Unavailable information is marked as such. eOGO, external compression of the outflow graft causing obstruction. Table S7: Laboratory parameters of eOGO patients who received percutaneous intervention. INR, International Normalized Ratio; LVAD, left ventricular assist device; eOGO, external compression of the outflow graft causing obstruction; LDH, lactate dehydrogenase; Hb, hemoglobin; aPTT, activated partial thromboplastin clotting time. Table S8: LVAD parameters of eOGO patients who received percutaneous intervention. LVAD, left ventricular assist device; eOGO, external compression of the outflow graft causing obstruction. Table S9: Follow‐up times and outcomes of eOGO patients with different treatments strategies. eO [file AOR-50-1060-s001.docx]

**Supplementary Material**

**
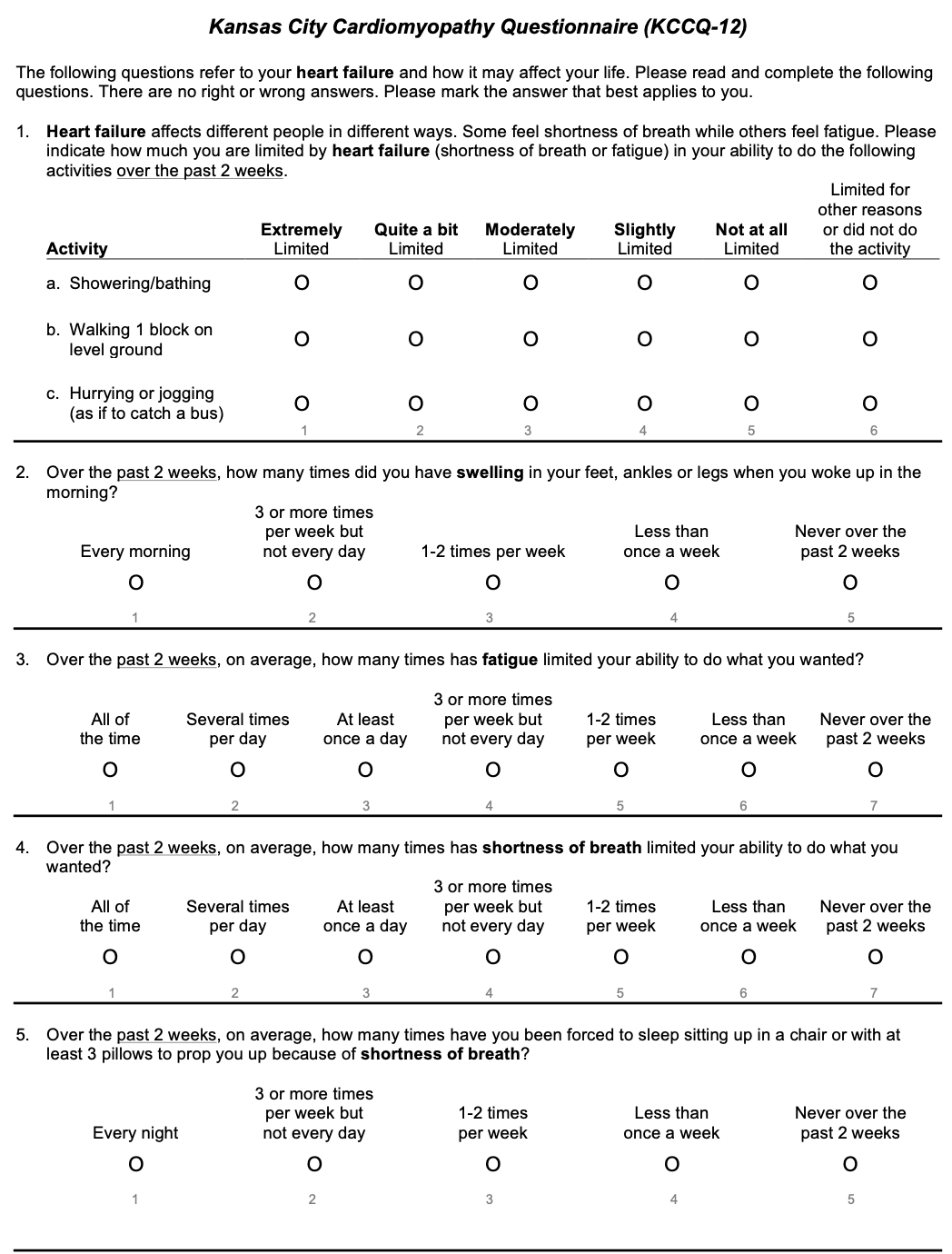
**

**
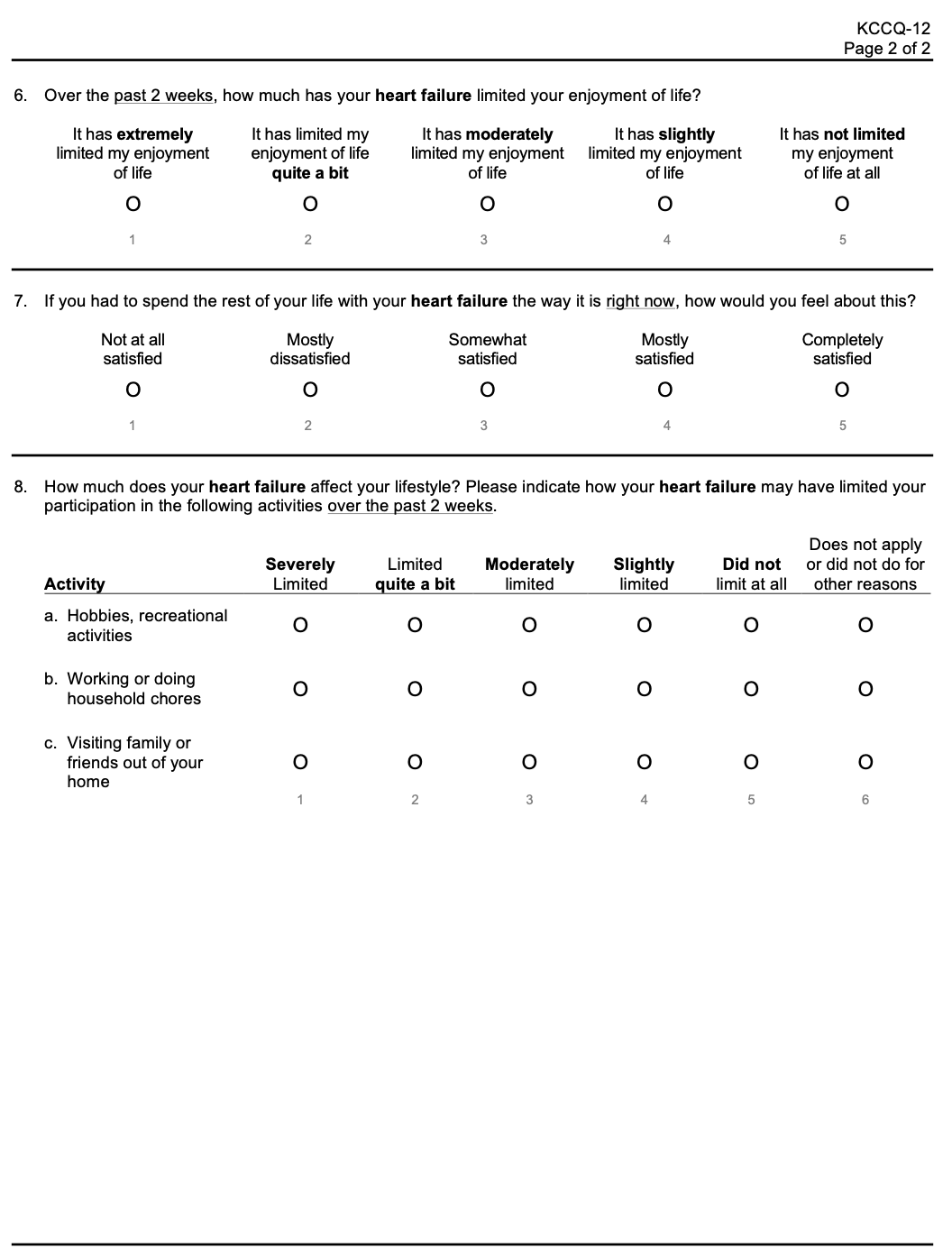
**

Figure S1: KCCQ-12 questionnaire used for quality-of-life assessment. *KCCQ-12*, short version of the Kansas City Cardiomyopathy Questionnaire.

| **Variable** | **Value** |
| --- | --- |
| Male, N (%) | 24 (77.4) |
| Age at HM3 implantation, mean ± SD | 57 ± 11.5 |
| Days on HM3 support, median [IQR] | 1219 [976, 1917] |
| Cardiac pathology, N (%) |  |
| Ischaemic cardiomyopathy | 18 (58.1) |
| Dilated cardiomyopathy | 12 (38.7) |
| Non-ischaemic cardiomyopathy | 1 (3.2) |
| Therapy target, N (%) |  |
| Destination | 23 (74.2) |
| Bridge to transplant | 8 (25.8) |
| Surgical access, N (%) |  |
| Full sternotomy | 26 (83.9) |
| Lateral thoracotomy | 5 (16.1) |

Table S1: Demographic and baseline characteristics of HM3 patients with eOGO at the implantation centre. Data on surgical access for implantation was available for nine patients and was taken into account for the percentage calculations. *HM3*, HeartMate 3; *eOGO*, external compression of the outflow graft causing obstruction.

| **Case** | **Sex** | **Age at LVAD implantation** | **Days on HM3 support** | **Age at diagnosis** | **Cardiac pathology** | **Therapy target** | **Surgical access** |
| --- | --- | --- | --- | --- | --- | --- | --- |
| 1 | Male | 34 | 1008 | 37 | DCMP | Bridge | Sternotomy |
| 2 | Female | 49 | 1560 | 54 | DCMP | Destination | Sternotomy |
| 3 | Male | 50 | 2220 | 57 | ICMP | Destination | Sternotomy |
| 4 | Female | 53 | 1900 | 58 | NICMP | Destination | Sternotomy |
| 5 | Male | 76 | 1135 | 80 | ICMP | Destination | Sternotomy |
| 6 | Male | 63 | 944 | 66 | ICMP | Destination | Sternotomy |
| 7 | Male | 46 | 1121 | 49 | DCMP | Bridge | Sternotomy |
| 8 | Male | 58 | 1763 | 63 | ICMP | Destination | Sternotomy |
| 9 | Male | 64 | 1222 | 67 | DCMP | Destination | Sternotomy |
| 10 | Male | 49 | 2434 | 56 | DCMP | Bridge | Sternotomy |
| 11 | Male | 61 | 1409 | 65 | DCMP | Destination | Sternotomy |
| 12 | Male | 40 | 2007 | 45 | DCMP | Destination | Sternotomy |
| 13 | Male | 71 | 784 | 73 | DCMP | Destination | Sternotomy |
| 14 | Female | 68 | 1049 | 71 | DCMP | Destination | Lateral thoracotomy |
| 15 | Male | 62 | 1934 | 67 | DCMP | Destination | Sternotomy |
| 16 | Male | 64 | 2413 | 70 | ICMP | Destination | Sternotomy |
| 17 | Male | 41 | 1539 | 45 | DCMP | Bridge | Sternotomy |
| 18 | Male | 58 | 1009 | 61 | ICMP | Destination | Sternotomy |
| 19 | Male | 64 | 1219 | 67 | ICMP | Destination | Sternotomy |
| 20 | Male | 64 | 2039 | 70 | ICMP | Destination | Sternotomy |
| 21 | Male | 46 | 1599 | 51 | ICMP | Bridge | Lateral thoracotomy |
| 22 | Female | 62 | 730 | 64 | ICMP | Bridge | Sternotomy |
| 23 | Male | 51 | 478 | 52 | ICMP | Destination | Lateral thoracotomy |
| 24 | Male | 71 | 3042 | 80 | ICMP | Destination | Sternotomy |
| 25 | Male | 66 | 889 | 69 | ICMP | Destination | Sternotomy |
| 26 | Female | 32 | 1131 | 35 | ICMP | Destination | Lateral thoracotomy |
| 27 | Female | 44 | 2255 | 50 | ICMP | Bridge | Sternotomy |
| 28 | Male | 71 | 163 | 71 | ICMP | Bridge | Lateral thoracotomy |
| 29 | Male | 66 | 624 | 67 | ICMP | Destination | Sternotomy |
| 30 | Female | 68 | 1096 | 71 | DCMP | Destination | Sternotomy |
| 31 | Male | 57 | 747 | 60 | ICMP | Destination | Sternotomy |

Table S2: Baseline characteristics of individual patients. *HM3*, HeartMate 3; *CMP*, cardiomyopathy; *ICMP*, ischaemic cardiomyopathy; *DCMP*, dilated cardiomyopathy; *NICMP*, non-ischaemic cardiomyopathy; *eOGO*, external compression of the outflow graft causing obstruction.

| **Years on LVAD support** | **Incidence** | **Numbers at risk** | **Cumulative incidence %** |
| --- | --- | --- | --- |
| 1 | 1 | 31 | 3.2 |
| 2 | 2 | 30 | 9.7 |
| 3 | 8 | 28 | 35.5 |
| 4 | 7 | 20 | 58.1 |
| 5 | 4 | 13 | 71.0 |
| 6 | 4 | 9 | 83.9 |
| 7 | 4 | 5 | 96.8 |
| 8 | 0 | 1 | 96.8 |
| 9 | 1 | 1 | 100.0 |

Table S3: Incidence table of patients on HM3 support who developed eOGO. *HM3*, HeartMate 3; *eOGO*, external compression of the outflow graft causing obstruction.

| **Symptom** | **N (%)** |
| --- | --- |
|  |  |
| Heart failure symptoms |  |
| Dyspnoea | 10 (32.3) |
| Low-flow alert | 6 (19.4) |
| Hydropic decompensation | 5 (16.1) |
| Angina pectoris | 3 (9.7) |
| Syncope | 1 (0.9) |
| Unspecific symptoms |  |
| Anaemia | 1 (3.2) |
| Fatigue | 1 (3.2) |
| Incidental finding | 7 (22.6) |

Table S4: Symptoms displayed by eOGO patients. *eOGO*, external compression of the outflow graft causing obstruction.

| **Case** | **Symptoms** | **Degree of stenosis in CT** | **eOGO severity** | **Treatment** |
| --- | --- | --- | --- | --- |
| 1 | Incidental finding | 80% | Severe | Stenting |
| 2 | Dyspnoea, hydropic decompensation | 50–60% | Moderate | Stenting |
| 3 | Dyspnoea | 40% | Mild | Stenting |
| 4 | Incidental finding | 50–75% | Moderate | Stenting |
| 5 | Dyspnoea, angina pectoris | >80% | Severe | Stenting |
| 6 | Dyspnoea | 75% | Severe | Stenting |
| 7 | Low-flow alert, dyspnoea | 80% | Severe | Stenting |
| 8 | Hydropic decompensation | 70% | Moderate | Stenting |
| 9 | Low-flow alert, anaemia | 70% | Moderate | Stenting |
| 10 | Incidental finding | 60% | Moderate | Stenting |
| 11 | Incidental finding | 50% | Moderate | Stenting |
| 12 | Incidental finding | Beginning jelly build-up | Mild | Stenting |
| 13 | Low-flow alert | Severe stenosis | Severe | Stenting |
| 14 | Low-flow alert | 75–85% | Severe | Stenting |
| 15 | Hydropic decompensation | 85% | Severe | Stenting |
| 16 | Angina pectoris | 60–70% | Moderate | Stenting |
| 17 | Fatigue | 50% | Moderate | Stenting |
| 18 | Hydropic decompensation | 75% | Severe | Stenting |
| 19 | Dyspnoea | 72–82% | Severe | Stenting |
| 20 | Hydropic decompensation | >50% | Moderate | Stenting |
| 21 | Low-flow alert | 80% | Severe | Surgery |
| 22 | Incidental finding | 50% | Moderate | Transplantation |
| 23 | Angina pectoris | 25% | Mild | Wait |
| 24 | Asymptomatic | No relevant stenosis | Mild | Wait |
| 25 | Low-flow alert, syncope | 50% | Moderate | Wait |
| 26 | Dyspnoea | 25% | Mild | Wait |
| 27 | Dyspnoea | 20% | Mild | Wait |
| 28 | Dyspnoea | Minimal jelly build-up | Mild | Wait |
| 29 | Dyspnoea | <20% | Mild | Wait |
| 30 | Incidental finding | 60% | Moderate | Wait |
| 31 | Incidental finding | Clinically irrelevant jelly build-up | Mild | Wait |

Table S5: Clinical symptoms, diagnostic assessment and treatment of individual patients. *CT*, computed tomography; *eOGO*, external compression of the outflow graft causing obstruction.

| **Case** | **Stent type** |
| --- | --- |
| 1 | 1x Smart Control: 14/80mm |
| 2 | unavailable |
| 3 | 2x Bentley BeGraft Aortic: 14/49mm, 14/59mm |
| 4 | 2x Bentley BeGraft Aortic: 14/59mm, 14/49mm |
| 5 | 2x Bentley BeGraft Aortic: 14/49mm |
| 6 | unavailable |
| 7 | 2x Smart Control: 14/80mm & 14/40 |
| 8 | 2x Bentley BeGraft Aortic: 14/59mm, 14/49mm |
| 9 | 2x Bentley BeGraft Aortic |
| 10 | 2x Bentley BeGraft Aortic: 14/59mm |
| 11 | Bentley BeGraft Aortic: 14/59mm |
| 12 | unavailable |
| 13 | 2x Bentley BeGraft Aortic: 14/60mm, 14/50mm |
| 14 | 2x Smart Control: 14/80mm |
| 15 | 3x Bentley BeGraft Aortic |
| 16 | 2x Bentley BeGraft Aortic: 14/59mm |
| 17 | 2x Bentley BeGraft Aortic |
| 18 | 3x Bentley BeGraft Aortic: 14/59mm |
| 19 | 3x Bentley BeGraft Aortic: 14/59mm |
| 20 | 2x Bentley BeGraft Aortic: 14/59mm, 14/49mm |

Table S6: Stent types used for eOGO patients during percutaneous intervention. Unavailable information is marked as such. *eOGO*, external compression of the outflow graft causing obstruction.

| **Case** | **LDH at admission** | **LDH at discharge** | **Hb at admission** | **Hb at discharge** | **INR at admission** | **INR at discharge** | **aPTT at admission** | **aPTT at discharge** |
| --- | --- | --- | --- | --- | --- | --- | --- | --- |
| 1 | 298 | 182 | 91.2 | 6.3 | 2.5 | 3.3 | 41 | 139 |
| 2 | 418 | 662 | 6.5 | 10.7 | 1.8 | 7 | 136.4 | 79.2 |
| 3 | 353 | 281 | 6.6 | 27.3 | 2.1 | 2 | 80.6 | 39.9 |
| 4 | - | 192 | - | 15.2 | - | 1.7 | - | 180 |
| 5 | 418 | 352 | 6.8 | 7 | 2.6 | 2.1 | 57 | 59.1 |
| 6 | 196 | - | 5.9 | - | 2.7 | 2.2 | 43.9 | 41 |
| 7 | 338 | - | 12.1 | - | 2.7 | 3.2 | 37.6 | 44.1 |
| 8 | 228 | 236 | 23.3 | - | 2.6 | 2.8 | 37 | 46 |
| 9 | 217 | 229 | 3.4 | 6.5 | 2 | 2.7 | 52 | 79 |
| 10 | 258 | 204 | 5.3 | - | 2.6 | 2.2 | 37.6 | 35.8 |
| 11 | 197 | - | 10.8 | - | 1.5 | 2.8 | 58.2 | 60 |
| 12 | 98 | 181 | 3.8 | - | 2.5 | 1.5 | 58.2 | 56.3 |
| 13 | 128 | 128 | - | 8.2 | 2.9 | 2.8 | 59.4 | 68.9 |
| 14 | 303 | 335 | 18.2 | - | 2.7 | 2.2 | 40 | 43 |
| 15 | 253 | 257 | 18 | - | 1.9 | 2.3 | 38.1 | 39.2 |
| 16 | 188 | 189 | 11.5 | - | 1.6 | 2.4 | 42 | 86.8 |
| 17 | 237 | 216 | 14.7 | - | 2.2 | 1.6 | 40.5 | 45.1 |
| 18 | 918 | 1650 | 11.9 | 24.1 | 7 | 2.9 | 60.5 | 76.8 |
| 19 | 282 | 258 | 25.5 | - | 2.7 | 2.1 | 26 | 36 |
| 31 | 264 | 299 | 9.3 | - | 2 | 2.5 | 41.2 | 43.9 |

Table S7: Laboratory parameters of eOGO patients who received percutaneous intervention. *INR*, International Normalized Ratio; *LVAD*, left ventricular assist device; *eOGO*, external compression of the outflow graft causing obstruction; *LDH,* lactate dehydrogenase; *Hb*, haemoglobin; *aPTT*, activated partial thromboplastin clotting time.

| **Case** | **Flow at admission (L/min)** | **Flow at discharge (L/min)** | **Speed at admission (rpm)** | **Speed at discharge (rpm)** | **Motor power at admission (W)** | **Motor power at discharge (W)** |
| --- | --- | --- | --- | --- | --- | --- |
| 1 | 4.5 | 4.1 | 5400 | 5400 | 3 | 3 |
| 2 | 3.1 | 5.6 | 5500 | 7400 | 4.1 | 7.7 |
| 3 | 3.8 | 4.2 | 5500 | 5500 | 4 | 4.1 |
| 4 | 4.6 | 5.3 | 5100 | 5100 | 4.5 | 3.5 |
| 5 | 4.5 | 4.8 | 5500 | 5550 | 4.2 | 4.2 |
| 6 | 4.6 | 5.3 | 5500 | 5500 | 4.1 | 4.2 |
| 7 | 3.6 | 5.5 | 5700 | 5700 | 4.2 | 4.6 |
| 8 | 4.3 | 4.7 | 5500 | 5500 | 4.3 | 4.2 |
| 9 | 4.1 | 4.7 | 5500 | 5500 | 4.3 | 4.2 |
| 10 | 4.6 | 4.5 | 5400 | 5400 | 4.1 | 4 |
| 11 | 4.1 | 4.4 | 5500 | 5500 | 4.1 | 4 |
| 12 | 4.5 | 3.6 | 5700 | 5200 | 4.5 | 3.8 |
| 13 | 4.1 | 4.1 | 5600 | 5600 | 4.5 | 4.1 |
| 14 | 2.4 | 4.3 | 5800 | 5600 | 3.9 | 4.1 |
| 15 | 5.7 | 5.5 | 5800 | 5899 | 4.5 | 2.6 |
| 16 | 5.3 | 5.2 | 5700 | 5700 | 4.3 | 4.4 |
| 17 | 5.9 | 6.1 | 6200 | 6200 | 5.4 | 5.5 |
| 18 | 5.8 | 5.5 | 5350 | 5900 | 3.9 | 4.5 |
| 19 | 4 | 5.7 | 6200 | 6200 | 5 | 5.4 |
| 20 | 4.7 | 4.2 | 5400 | 5450 | 4.2 | 4.1 |

Table S8: LVAD parameters of eOGO patients who received percutaneous intervention. *LVAD*, left ventricular assist device; *eOGO*, external compression of the outflow graft causing obstruction.

| **Case** | **Treatment** | **Follow-up time (days)** | **Outcome** |
| --- | --- | --- | --- |
| 1 | Stenting | 1965 | Ongoing |
| 2 | Stenting | 7 | Deceased |
| 3 | Stenting | 829 | Ongoing |
| 4 | Stenting | 1057 | Ongoing |
| 5 | Stenting | 434 | Ongoing |
| 6 | Stenting | 40 | Deceased |
| 7 | Stenting | 1094 | Ongoing |
| 8 | Stenting | 919 | Deceased |
| 9 | Stenting | 85 | Deceased |
| 10 | Stenting | 1017 | Ongoing |
| 11 | Stenting | 752 | Ongoing |
| 13 | Stenting | 1023 | Ongoing |
| 13 | Stenting | 806 | Deceased |
| 14 | Stenting | 2156 | Ongoing |
| 15 | Stenting | 1018 | Ongoing |
| 16 | Stenting | 864 | Ongoing |
| 17 | Stenting | 666 | Ongoing |
| 18 | Stenting | 2 | Deceased |
| 19 | Stenting | 686 | Ongoing |
| 20 | Stenting | 802 | Ongoing |
| 21 | Surgery | 522 | Deceased |
| 22 | Transplantation | 1160 | Ongoing |
| 23 | Wait | 595 | Ongoing |
| 24 | Wait | 302 | Ongoing |
| 25 | Wait | 267 | Deceased |
| 26 | Wait | 1003 | Ongoing |
| 27 | Wait | 612 | Ongoing |
| 28 | Wait | 339 | Deceased |
| 29 | Wait | 514 | Ongoing |
| 30 | Wait | 558 | Ongoing |
| 31 | Wait | 459 | Ongoing |

Table S9: Follow-up times and outcomes of eOGO patients with different treatments strategies. *eOGO*, external compression of the outflow graft causing obstruction.

| **Case** | **Q.1a** | **Q.1b** | **Q.1c** | **Q.2** | **Q.3** | **Q.4** | **Q.5** | **Q.6** | **Q.7** | **Q.8a** | **Q.8b** | **Q.8c** | **Health status** |
| --- | --- | --- | --- | --- | --- | --- | --- | --- | --- | --- | --- | --- | --- |
| 3 | 5 | 4 | 1 | 5 | 5 | 5 | 5 | 2 | 4 | 4 | 4 | 5 | fair-good |
| 4 | 1 | 5 | 1 | 5 | 5 | 7 | 5 | 5 | 5 | 5 | 1 | 5 | fair-good |
| 5 | 4 | 2 | 1 | 1 | 2 | 2 | 5 | 1 | 1 | 1 | 3 | 2 | very poor-poor |
| 7 | 2 | 2 | 1 | 5 | 3 | 4 | 2 | 3 | 2 | 3 | 2 | 4 | poor-fair |
| 10 | 4 | 5 | 4 | 5 | 7 | 7 | 5 | 5 | 3 | 5 | 3 | 5 | good-excellent |
| 14 | 2 | 3 | 2 | 5 | 7 | 3 | 5 | 5 | 5 | 5 | 4 | 5 | good-excellent |
| 15 | 2 | 2 | 1 | 4 | 2 | 2 | 5 | 3 | 3 | 1 | 2 | 3 | poor-fair |
| 19 | 4 | 3 | 1 | 5 | 3 | 3 | 5 | 4 | 3 | 1 | 4 | 3 | fair-good |
| 22 | 1 | 1 | 1 | 1 | 7 | 4 | 5 | 2 | 4 | 1 | 2 | 4 | poor-fair |
| 23 | 1 | 1 | 1 | 1 | 2 | 7 | 5 | 2 | 2 | 5 | 1 | 5 | poor-fair |
| 26 | 1 | 1 | 1 | 5 | 1 | 1 | 2 | 5 | 1 | 3 | 4 | 1 | poor-fair |
| 27 | 5 | 3 | 1 | 5 | 7 | 7 | 5 | 5 | 5 | 5 | 5 | 5 | good-excellent |
| 29 | 2 | 3 | 1 | 5 | 7 | 7 | 5 | 4 | 3 | 5 | 5 | 5 | fair-good |
| 30 | 5 | 4 | 1 | 5 | 2 | 2 | 5 | 3 | 3 | 1 | 1 | 2 | poor-fair |
| 31 | 5 | 5 | 3 | 5 | 7 | 7 | 5 | 5 | 3 | 3 | 2 | 5 | good-excellent |

Table S10: Results from quality-of-life assessment of available eOGO patients post-treatment. The KCCQ-12 was used to interview patients with the question number and corresponding score outlined as well as the resulting health status. *eOGO*, external compression of the outflow graft causing obstruction, *KCCQ-12*, short version of the Kansas City Cardiomyopathy Questionnaire; *Q*, question.
